# The Disulfide Stress Response and Protein *S*-thioallylation Caused by Allicin and Diallyl Polysulfanes in *Bacillus subtilis* as Revealed by Transcriptomics and Proteomics

**DOI:** 10.3390/antiox8120605

**Published:** 2019-11-29

**Authors:** Bui Khanh Chi, Nguyen Thi Thu Huyen, Vu Van Loi, Martin Clemens Horst Gruhlke, Marc Schaffer, Ulrike Mäder, Sandra Maaß, Dörte Becher, Jörg Bernhardt, Miriam Arbach, Chris J. Hamilton, Alan J. Slusarenko, Haike Antelmann

**Affiliations:** 1 Institute of Biology-Microbiology, Freie Universität Berlin, D-14195 Berlin, Germany; nve1@cdc.gov (B.K.C.); huyen308@gmail.com (N.T.T.H.); vu.v.loi@fu-berlin.de (V.V.L.); joerber@uni-greifswald.de (J.B.); 2 Division of Global Health Protection, Center for Global Health, Center for Disease Control and Prevention, Ngo Quyen Str. 2, Hanoi 100000, Vietnam; 3 Faculty of Biotechnology, Ho Chi Minh University of Food Industry, Ho Chi Minh City 700000, Vietnam; 4 Department of Plant Physiology, RWTH Aachen University, D-52056 Aachen, Germany; Martin.Gruhlke@rwth-aachen.de (M.C.H.G.); alan.slusarenko@bio3.rwth-aachen.de (A.J.S.); 5 Interfaculty Institute for Genetics and Functional Genomics, University Medicine Greifswald, D-17489 Greifswald, Germany; marc.schaffer@uni-greifswald.de (M.S.); ulrike.maeder@uni-greifswald.de (U.M.); 6 Institute of Microbiology, University of Greifswald, D-17489 Greifswald, Germany; sandra.maass@uni-greifswald.de (S.M.); dbecher@uni-greifswald.de (D.B.); 7 School of Pharmacy, University of East Anglia, Norwich Research Park, Norwich NR4 7TJ, UK; miriam.arbach@posteo.de (M.A.); C.Hamilton@uea.ac.uk (C.J.H.); 8 ECOspray Ltd., Grange Farm, Hilborough, Thetford IP26 5BT, UK

**Keywords:** *Bacillus subtilis*, allicin, diallyl polysulfane, bacillithiol, *S*-thioallylation

## Abstract

Garlic plants (*Allium sativum* L.) produce antimicrobial compounds, such as diallyl thiosulfinate (allicin) and diallyl polysulfanes. Here, we investigated the transcriptome and protein *S*-thioallylomes under allicin and diallyl tetrasulfane (DAS4) exposure in the Gram-positive bacterium *Bacillus subtilis*. Allicin and DAS4 caused a similar thiol-specific oxidative stress response, protein and DNA damage as revealed by the induction of the OhrR, PerR, Spx, YodB, CatR, HypR, AdhR, HxlR, LexA, CymR, CtsR, and HrcA regulons in the transcriptome. At the proteome level, we identified, in total, 108 *S*-thioallylated proteins under allicin and/or DAS4 stress. The *S*-thioallylome includes enzymes involved in the biosynthesis of surfactin (SrfAA, SrfAB), amino acids (SerA, MetE, YxjG, YitJ, CysJ, GlnA, YwaA), nucleotides (PurB, PurC, PyrAB, GuaB), translation factors (EF-Tu, EF-Ts, EF-G), antioxidant enzymes (AhpC, MsrB), as well as redox-sensitive MarR/OhrR and DUF24-family regulators (OhrR, HypR, YodB, CatR). Growth phenotype analysis revealed that the low molecular weight thiol bacillithiol, as well as the OhrR, Spx, and HypR regulons, confer protection against allicin and DAS4 stress. Altogether, we show here that allicin and DAS4 cause a strong oxidative, disulfide and sulfur stress response in the transcriptome and widespread *S*-thioallylation of redox-sensitive proteins in *B. subtilis*. The results further reveal that allicin and polysulfanes have similar modes of actions and thiol-reactivities and modify a similar set of redox-sensitive proteins by *S*-thioallylation.

## 1. Introduction

Garlic (*Allium sativum*) has been historically used as a medicinal plant for the treatment of infectious diseases, such as tuberculosis, due to the production of volatile reactive sulfur compounds. The main thiol-reactive sulfur ingredient of garlic is the diallyl thiosulfinate (allicin), which acts as antimicrobial and inhibits the growth and survival of several important human pathogens, including multi-drug resistant bacteria, fungi, and parasites [1,2,3,4,5,6,7,8,9]. Upon garlic tissue damage, the enzyme cysteine-*S*-lyase alliinase is released from the vacuole into the cytosol to synthesize allyl sulfenic acid and dehydroalanine from the odor-less precursor alliin. Allicin forms by spontaneous condensation of two allyl sulfenic acid molecules [5,10,11]. Allicin decomposition occurs rapidly during heating or distillation resulting in various diallyl polysulfanes, with 2–6 sulfur atoms organized as sulfur chains, including diallyl di-, tri-, tetra-, penta-, and hexa polysulfanes (DAS2-6) [3,4]. DAS2 and DAS3 are the most frequently observed sulfur compounds present in garlic oils [12,13,14]. 

Allicin and diallyl polysulfanes DAS2-6 showed different extents of thiol-reactivity and microbicidal effects, with DAS4-6 and allicin being more potent antimicrobials compared to diallyl di- and trisulfides (DAS2-3) [3,9]. The effects of garlic polysulfanes DAS2-6 were compared on the growth and the minimal inhibitory concentration (MIC) of *Bacillus subtilis*, *Helicobacter pylori*, *Staphylococcus aureus*, and fungi, which showed that the antimicrobial activity of polysulfanes increases with longer sulfur chain length [9,15,16,17]. However, the stability of DAS5-6 is also strongly decreased compared to DAS2-4, and polysulfanes of longer chain length tend to rearrange to a mix of shorter polysulfanes when incubated with glutathione (GSH) in vitro [9,18].

The thiol-reactive modes of actions of allicin and diallyl polysulfanes and their reactions with low molecular weight (LMW) thiols have been investigated in *Escherichia coli*, *S. aureus*, and *B. subtilis* [7,9,19]. The mode of action of garlic sulfur compounds was attributed to *S*-thioallylation of LMW and protein thiols, causing rapid depletion of the reduced thiol pool upon allicin and DAS4 stress [5,7,8,19,20]. Treatment of GSH with allicin and polysulfanes in vitro results in a thiol-disulfide exchange reaction and the formation of GSH conjugated *S*-thioallylated di-, tri-, and tetra-sulfanes, and allyl thiols or allyl persulfides [3,9,13]. The *S*-thioallylated di- and trisulfanes were the major GSH conjugates measured upon GSH exposure to DAS3-6 in vitro [9]. In contrast to *E. coli* and eukaryotes, the Gram-positive bacterium *B. subtilis* does not utilize GSH, but instead produces bacillithiol (BSH) as alternative LMW thiol [21,22,23]. In *B. subtilis*, the depletion of the BSH and cysteine by the polysulfanes DAS3-4 occurred rapidly and was accompanied by formation of allyl thiols as polysulfane detoxification products [9]. GSH and BSH were further important for protection against allicin and diallyl polysulfane stress in yeast, *B. subtilis*, and *S. aureus* [5,9,19,24,25]. 

In *E. coli*, *B. subtilis*, *S. aureus*, and human cells, allicin and polysulfanes also caused increased protein *S*-thioallylation to deplete the cellular pool of protein thiols, leading to inactivation of proteins and cell death [7,8,19,20,26]. The inactivation of enzymes by *S*-thioallylation has been shown for the cysteine protease papain, alcohol dehydrogenases, enolase, isocitrate lyase, and glyceraldehyde-3-phosphate dehydrogenase GapDH in vitro [7,8,19,26]. In the human Jurkat cell proteome, about 332 proteins were modified by *S*-thioallylations after allicin stress [26]. In *E. coli* cells, about 90 *S*-thioallylated peptides in 73 proteins were identified that include mostly high abundant proteins [7,8,20]. In *S. aureus*, 57 proteins were identified as targets for *S*-thioallylation in *S. aureus*, including metabolic and redox enzymes as well as redox-sensing regulators of the MarR/SarA family [19]. We could further unravel the pathways for regeneration from allicin stress in *S. aureus*. While BSH and the disulfide reductase MerA were shown to function in allicin detoxification, the BrxA/BSH/YpdA pathway catalyzed the removal of *S*-thioallylation from BSH and proteins to restore the thiol-redox homeostasis [19]. 

In *B. subtilis*, BSH participates in redox modification of Cys residues under HOCl stress, leading to widespread *S*-bacillithiolation, which functions in thiol-protection and redox regulation of proteins similarly as *S*-glutathionylation [21,27,28,29,30,31,32,33,34,35]. Among the targets for *S*-bacillithiolation are the translation elongation factor TufA, the methionine synthase MetE, the inosine monophosphate dehydrogenase GuaB, and the inorganic pyrophosphatase PpaC [27,30,35]. *S*-bacillithiolation of MetE occurs at its Zn-binding active site, leading to its inactivation and methionine auxotrophy in HOCl-treated cells [27,35]. The redox-sensing OhrR repressor is inhibited by *S*-bacillithiolation under HOCl and cumene hydroperoxide (CHP) stress, resulting in derepression of the *ohrA* peroxiredoxin, which confers resistance to the oxidants [27,29,36]. However, it is unknown whether garlic sulfur compounds modify similar targets by *S*-thioallylation in *B. subtilis*. 

Here, we aimed to investigate the regulatory stress responses and targets for *S*-thioallylations in response to allicin and diallyl tetrasulfane (DAS4) in *B. subtilis*. Both sulfur compounds allicin and DAS4 were shown to elicit a similar strong thiol-specific oxidative and sulfur stress response in the transcriptome of *B. subtilis*. About 108 targets for *S*-thioallylation were identified by shotgun proteomics, including the majority of previously identified *S*-thiolated proteins under HOCl stress, such as TufA, MetE, YxjG, GuaB, SerA, and PpaC, as well as the redox-sensing regulators OhrR, HypR, YodB, and CatR. Growth comparisons revealed that BSH and the OhrR, PerR, HypR, and Spx regulons contribute to allicin protection mechanisms in *B. subtilis*. 

## 2. Materials and Methods 

### 2.1. Bacterial Strains and Growth Conditions 

*B. subtilis* strains used in this study are derivatives of the parent strains 168 (*trpC2*), JH642 (*trpC2* attSPß), and CU1065 (*trpC2* pheA1), which were cultivated at 37 °C in Belitsky minimal medium (BMM) as described previously [37]. *B. subtilis* mutant strains used for phenotype studies include Δ*spx* (*trpC2*,*spx*::*neo*^r^) [38], Δ*ohrA* (*trpC2*, *ohrA*::cm^r^) [27], Δ*hypR* (*trpC2*, *hypR*::*cm*^r^) [39], HB9121 (CU1065 *trpC2*, *ohrR*::km^r^ *ohrR*-FLAG (Spc^r^) *ohrA-cat lacZ* (Neo^r^) [29], and HB11002 (CU1065 *trpC2*, *bshA*::mls^r^) [28]. Antibiotics were supplemented when required for the mutants at the following doses: 1 µg/mL erythromycin, 25 µg/mL lincomycin, 5 µg/mL chloramphenicol, 10 µg/mL kanamycin, 100 µg/mL spectinomycin. 

For the stress experiments with allicin and DAS4, cells were grown in BMM to an optical density at 500 nm (OD_500_) of 0.4 and exposed to sub-lethal doses of 90 and 250 µM allicin and 92 µM DAS4. The statistics of significant changes in the growth curves was determined using the Student’s unpaired two-tailed t-test by the GraphPad Prism software. Allicin was synthetized by oxidation of 3-[prop-2-en-1-yl) disulfanyl] prop-1-ene (diallyl disulfide) with peracetic acid, as described [24]. DAS4 was synthesized and purified, as previously described [9]. 

### 2.2. Identification of S-Thioallylated Proteins Using LTQ-Orbitrap Mass Spectrometry

*B. subtilis* 168 was grown in BMM and treated with 90 µM allicin and 92 µM DAS4 for 30 min, followed by harvesting of cells, and alkylation in *N*-ethylmaleimide (NEM) buffer, as described [26,27]. NEM-alkylated protein extracts were subjected to tryptic in-gel-digestion and LTQ Orbitrap Velos mass spectrometry, as described [27]. *S*-thioallylated proteins were identified by searching all tandem mass spectrometry (MS/MS) spectra against the *B. subtilis* 168 target-decoy protein sequence database extracted from UniprotKB release 12.7 (UniProt Consortium, Nucleic acids research 2007, 35, D193-197) using Sorcerer^TM^-SEQUEST^®^ (Sequest v. 2.7 rev.11, Thermo Electron, including Scaffold 4.0; Proteome Software, Inc., Portland, OR, USA). The SEQUEST search was carried out with the previously used parameters [27], including a parent ion mass tolerance of 10 ppm and a fragment ion mass tolerance of 1.00 Da. Up to two tryptic mis-cleavages were allowed. Methionine oxidation (Met+15.994915 Da), cysteine alkylation by *N*-ethylmaleimide (Cys+125.04767 Da), and cysteine *S*-thioallylation by allicin (Cys+72.00337 Da for C_3_H_5_S_1_) were set as variable modifications. The mass spectrometry data have been deposited to the ProteomeXchange Consortium via the PRIDE partner repository [40,41] with the dataset identifier PXD013607. 

### 2.3. Microarray Transcriptome Analysis 

For microarray analysis, *B. subtilis* wild-type cells were grown in minimal medium to OD_500_ of 0.4 and harvested before and 30 min after treatment with 90 µM allicin and 92 µM DAS4. Total RNA was isolated by the acid phenol method as described [42]. For transcriptome analysis, 35 µg RNA were DNase-treated using the RNase-Free DNase Set (Qiagen, Hilden, Germany) and purified using the RNA Clean-Up and Concentration Kit (Norgen Biotek, Thorold, ON, Canada). The quality of the RNA preparations was assessed by means of the Agilent 2100 Bioanalyzer (Agilent Technologies, Waldbronn, Germany). Fluorescently labeled cDNA was synthesized and purified as described previously [27,43]. The allicin and DAS4 samples were labeled with Cy5, and the control samples were labeled with Cy3. 600 ng of Cy5- and Cy3-labeled cDNA were co-hybridized in a 1:1 ratio with the microarray based on the instruction of Agilent’s protocol (Two-Color Microarray-based Gene Expression Analysis, version 5.5, Agilent Technologies, Waldbronn, Germany). Data were extracted and processed using the feature extraction software (version 10.5, Agilent Technologies, Waldbronn, Germany). The error-weighted average of the log ratios of the probes was calculated for each gene using the Rosetta Resolver software (version 7.2.1, Rosetta Biosoftware, Seattle, WA, USA). Normalization was applied to log ratios by using to the Lowess algorithm. Genes showing induction or repression ratios of at least three-fold in two independent biological replicates were considered as significantly induced and subsets of the most interesting regulons are displayed in the Voronoi transcriptome treemap. All transcriptional fold-changes and log2 fold changes of the protein-coding genes and other RNA features quantified for DAS4 or allicin stress versus the control samples including the standard deviations and coefficient of variations are listed in Tables S1 and S2. The microarray datasets are available in NCBI’s gene expression omnibus (GEO) database under accession number [GSE132981].

### 2.4. Construction of the Voronoi Transcriptome Treemap 

For construction of the allicin and DAS4 transcriptome treemaps, the Paver software (DECODON GmbH, Greifswald, Germany) was applied [44]. The treemap visualizes the log2 fold-changes of highly upregulated redox regulons under allicin and DAS4 stress using a red–blue color gradient. Regulons are indicated with larger white labels, genes and operons are shown with smaller labels. The cell size is defined as ratio of expression levels under allicin treatment relative to the control. 

### 2.5. Immunoprecipitation (IP) and Non-Reducing SDS-PAGE Analysis of OhrR-FLAG, HypR, YodB, and CatR Proteins

The OhrR-FLAG protein expressing *B. subtilis* strain HB9121 was grown in BMM and exposed to 90 µM allicin at an OD_500_ of 0.4. Cells were harvested before (as untreated control) and 30 min after allicin stress in TE-buffer (10 mM Tris-HCl, pH8; 1 mM EDTA) with 100 mM iodoacetamide. Alkylated protein extracts were used for IP of OhrR-FLAG protein using anti-FLAG M2-affinity agarose (Invitrogen) according to the instructions of the manufacturer. For IP of HypR, YodB, and CatR, protein extracts of allicin-treated cells were subjected to Dynabead Protein A sepharose coupled to polyclonal HypR, YodB, and CatR antibodies, as described previously [27,45]. The precipitated OhrR-FLAG, HypR, YodB, and CatR proteins were eluted by boiling in non-reducing SDS sample buffer (4% SDS; 62.5 mM Tris-HCl pH 8.0, glycerol) and separated using 15% non-reducing SDS-PAGE. The protein bands were cut from the SDS-gel, tryptic in-gel digested, and the peptides analyzed by Orbitrap mass spectrometry as described above.

## 3. Results

### 3.1. Determination of Sub-Lethal Allicin and DAS4 Concentrations and Allicin Priming Assays in B. subtilis

**Figure 1:**
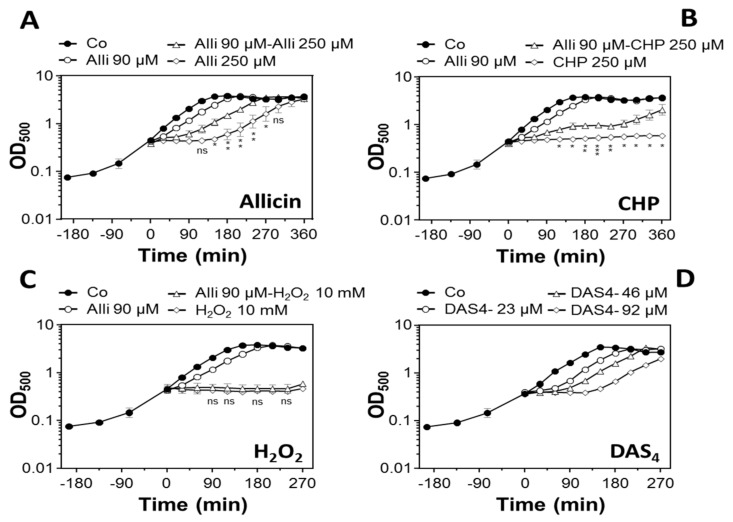
Growth curves of *B. subtilis* 168 wild type with different doses of allicin and DAS4 and allicin priming experiments for improved allicin and cumene hydroperoxide (CHP) resistance. (**A**) For allicin priming experiments, *B. subtilis* 168 was grown in Belitsky minimal medium (BMM) to an OD_500_ of 0.4 and exposed to 90 µM allicin for 30 min before subsequent treatment with the higher dose of 250 µM allicin. The growth was improved in the allicin-primed cells compared to non-primed cells, which were treated only with 250 µM allicin. (**B**) For allicin-CHP cross priming experiments, allicin-primed cells were exposed subsequently to lethal 250 µM CHP stress. Growth was improved in allicin-primed cells compared to non-primed cells. (**C**) For allicin-H_2_O_2_ cross priming, allicin-primed cells were treated with lethal 10 mM H_2_O_2_, but allicin priming did not improve growth. (**D**) *B. subtilis* 168 was exposed to different doses of 23, 46, and 92 µM DAS4 leading to different lag phases in growth and recovery after different times. The results are from three biological replicates. Error bars represent the standard deviations (SD) and the statistics was calculated using the Student’s unpaired two-tailed t-test by the GraphPad Prism software. The *p*-values for Alli 90 µM-Alli 250 µM versus Alli 250 µM (A) are *p* = 0.07 at 120 min; *p* = 0.026 at 150 min; *p* = 0.004 at 180 min; *p* = 0.005 at 210 min; *p* = 0.007 at 240 min; *p* = 0.032 at 270 min. For Alli 90 µM-CHP 250 µM versus CHP 250 µM (B), the *p*-values are *p* = 0.015 at 120 min; *p* = 0.013 at 150 min; *p* = 0.0036 at 180 min; *p* = 0.0009 at 210 min; *p* = 0.061 at 240 min; *p* = 0.0165 at 270 min. Symbols are ^ns^ *p* > 0.05, * *p* ≤ 0.05, ** *p* ≤ 0.01, and *** *p* ≤ 0.001.

First, we analyzed the growth of *B. subtilis* wild type cells after treatment with allicin and diallyl tetrasulfide (DAS4) to determine sub-lethal concentrations. Exposure of exponentially growing *B. subtilis* cells to 90 µM and 250 µM allicin resulted in a dose-dependent lag of growth for 20 min and 2 h, respectively, followed by rapid resumption of growth with the same rate as the untreated control (Figure 1A). This indicates that *B. subtilis* cells are able to recover fast in growth, presumably due to rapid detoxification of allicin and DAS4. We were further interested whether low doses of allicin can prime *B. subtilis* cells to mediate protection against subsequent higher doses of allicin or other oxidants. Indeed, priming of *B. subtilis* cells with 90 µM allicin resulted in protection against subsequent treatment with lethal 250 µM allicin, as shown by the faster growth recovery in primed cells compared to those treated with 250 µM allicin alone (Figure 1A). In addition, allicin primed *B. subtilis* cells could recover from lethal oxidative stress provoked by 250 µM CHP (Figure 1B). However, allicin priming did not confer cross-protection to lethal doses of 10 mM H_2_O_2_ (Figure 1C). Thus, allicin priming mediates protection against higher allicin doses and strong oxidants, such as CHP in *B. subtilis*. Next, we analyzed the growth of *B. subtilis* after exposure to different doses of DAS4. Treatment of *B. subtilis* with 23, 46, and 92 µM DAS4 also caused a dose-dependent growth delay of 1–2 h, followed by fast recovery of growth (Figure 1D), similarly as measured after allicin stress (Figure 1A). These growth profiles after allicin and DAS4 stress seem to indicate a disulfide stress response and are very similar, as shown previously for diamide and NaOCl stress in *B. subtilis* and for allicin in *E. coli* [7,27,46].

### 3.2. Allicin and DAS4 Cause a Strong Thiol-Specific Oxidative, Disulfide, and Sulfur Stress Response in the Transcriptome of B. subtilis 

**Figure 2:**
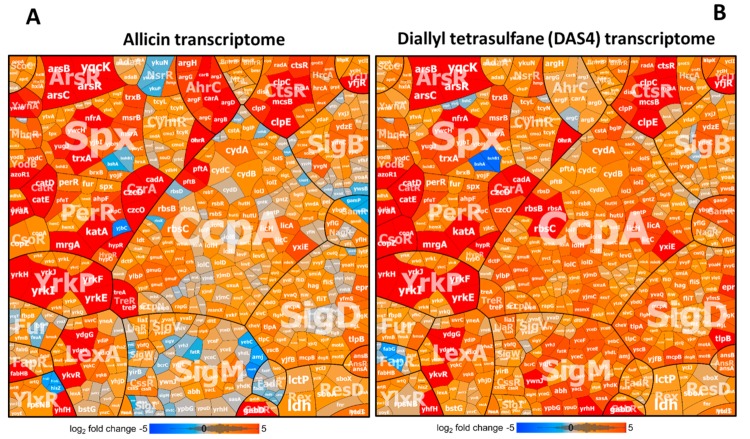
Allicin and DAS4 cause a strong thiol-specific oxidative and sulfur stress response in the transcriptome of *B. subtilis*, as revealed in the Voronoi transcriptome treemaps. The transcriptome treemap visualizes the log2 fold-changes in *B. subtilis* 168 cells in response to 90 µM allicin (**A**) and 92 µM DAS4 (**B**) as compared to the untreated controls. Genes are classified into operons and regulons based on RegPrecise and Subtiwiki databases. Log2 fold changes of gene expressions are indicated by the red–blue color code (red induction, blue repression) under allicin and DAS4 stress. Allicin and DAS4 both provoke a thiol-specific oxidative stress signature, as revealed by up-regulation of the OhrR, PerR, Spx, HypR, YodB, CatR, CtsR, and CymR regulons. The metal sensing CsoR, ArsR, and CzrA regulons were further induced. All fold-changes of gene expressions were quantified using microarrays and the data is listed in Tables S1 and S2.

To investigate in more detail the allicin- and DAS4-induced disulfide stress responses, *B. subtilis* was exposed to sub-lethal doses of 90 µM allicin or 92 µM DAS4 for 30 min and the changes in the transcriptome were analyzed using DNA microarrays, as described [27]. In total, 515 and 616 genes were reproducibly >3-fold up-regulated in the transcriptomes under allicin and DAS4 stress, respectively, in 2 biological replicates (Tables S1 and S2). The genes were sorted into regulons and subsets of the most strongly induced regulons under allicin and DAS4 stress are displayed in Voronoi transcriptome treemaps (Figure 2A,B, Tables S1 and S2). In general, both allicin and DAS4 caused a very similar stress response in the transcriptomes of *B. subtilis*. The OhrR, Spx, PerR, HypR, YodB, CatR, AdhR, ArsR, CzrA, CsoR, and CtsR regulons were most strongly induced under allicin and DAS4 stress, indicating that allicin and polysulfanes elicit a strong thiol-specific oxidative, disulfide and metal stress response, as well as protein damage (Figure 2A,B). Thus, these allicin and DAS4 expression profiles are similar compared to the NaOCl transcriptome signature in *B. subtilis* [27]. 

The OhrR-controlled *ohrA* peroxiredoxin gene was among the most highly induced genes under allicin and DAS4 stress (log2 fold-changes 6.4–8.7) (Tables S1 and S2). The OhrR repressor is redox-controlled by *S*-bacillithiolation under CHP and HOCl stress, leading to derepression of *ohrA*, which confers resistance to organic hydroperoxides and HOCl [27,29,47,48]. These results suggest that OhrR could sense allicin and DAS4 via *S*-thioallylation of its lone redox-sensing Cys22 residue. Furthermore, the PerR regulon was up-regulated under allicin and DAS4 stress, including the genes for catalase *katA* (5.8–7.8 log2 fold-changes), peroxiredoxins *ahpCF* (3.8–4.8-fold), and the miniferritin *mrgA* (5.5–8.6), which are indicative of an oxidative stress response. The PerR regulon is also induced by other disulfide stress conditions, such as NaOCl and diamide [27,46]. The majority of genes controlled by the disulfide stress specific Spx transcription factor displayed elevated expression under allicin and DAS4 exposure. These include several genes for thiol-disulfide oxidoreductases (*nfrA*, *yugJ*, *ywcH* and *yjbH*), thioredoxin/ thioredoxin reductase *(trxA, trxB*), methionine sulfoxide reductases (*msrA, msrB)* and other redox enzymes that are required to maintain cellular redox homeostasis [49,50] and displayed log2 fold-changes of 4.2–5.8 under allicin and DAS4 (Figure 2A,B, Tables S1 and S2). These redox enzymes could be involved in detoxification of allicin and polysulfanes or reduction of *S*-thioallylations to restore the BSH redox balance and reduced protein thiols. In addition, we noted the log2-fold changes of 1.5–3 for the Spx regulon genes *bshB2*, *bshC, brxA*, *brxB*, and *ypdA,* which encode the pathways for BSH biosynthesis, reduction of bacillithiol disulfide, and regeneration of *S*-thiolated proteins [19,28,51,52]. Similarly, the CymR regulon for cysteine biosynthesis was weakly induced (log2 fold changes of 1–2) by allicin and DAS4 (Tables S1 and S2). The induction of the genes for BSH and Cys biosynthesis supports the depletion of these LMW thiols in *B. subtilis*.

Similarly, as shown for HOCl and diamide stress, allicin and DAS4 resulted in up-regulation of regulons controlled by the redox-sensitive regulators HypR YodB, CatR and HxlR in *B. subtilis* (Figure 2A,B, Tables S1 and S2). The MarR/DUF24-family regulators HypR, YodB, and CatR were shown to sense HOCl, quinones, and diamide via conserved *N*-terminal Cys residues, while HxlR is more specific to control aldehydes detoxification [45,47,53,54,55,56]. Among the genes of the HypR, YodB, and CatR regulons, *hypR*, *hypO*, *azoR1, yodC*, and the *catDE* operon were most highly induced under allicin and DAS4 with log2-fold changes of 3.2–7.9 (Tables S1 and S2). The azoreductase AzoR1 and nitroreductase YodC function in diamide and quinone detoxification and could similarly contribute to allicin and polysulfane degradation [45,54]. The aldehyde-sensing HxlR and AdhR regulons, including the *hxlAB* operon for formaldehyde fixation [56] and *yraA* encoding a DJ1-family cysteine protease [57], showed log2-fold changes of 2.5–5.2 under allicin and polysulfanes. We have shown that the *hypR_Sa_-merA* operon of *S. aureus* USA300 was most strongly up-regulated under HOCl, diamide, and allicin stress [19,58]. The NADPH-dependent flavin disulfide reductase MerA was shown to be involved in allicin detoxification and conferred protection against allicin stress [19]. The Rrf2-family regulator YwnA is a close homolog of HypR of *S. aureus* [58]. Interestingly, *ywnA* and *ywnB*, encoding a *merA*-homologous flavin disulfide reductase, showed log2-fold changes of 4.6–6 in the allicin transcriptome of *B. subtilis* (Tables S1 and S2). This suggests similar functions of YwnB in allicin reduction in *B. subtilis*. 

In addition, allicin and DAS4 caused elevated transcription of the arsenate and copper responsive CzrA, ArsR, and CsoR regulons, controlling the metal ion efflux systems *czcDO*, *cadA*, *arsBC*, and *copZA* with log2-fold changes of 4.2–10.2 (Figure 2A,B, Tables S1 and S2) [59,60,61]. The metal binding Cys residues of the CzrA, ArsR, and CsoR repressors might be inactivated by *S*-thioallylation under allicin and DAS4 stress. 

Furthermore, the heat-shock CtsR regulon was highly induced under allicin and DAS4 treatment in *B. subtilis*. CtsR controls the ATP-dependent Clp protease subunits encoded by the *ctsR-mcsA-mcsB-clpC* operon, *clpE, clpP*, and *clpX* (log2 fold-changes of 6–9.5). The HrcA regulon, regulating heat-specific chaperones of the *dnaJK-grpE-hrcA* and *groESL* operons, was also up-regulated by allicin and DAS4 (log2 fold changes of ~2.7–4) (Figure 2A,B, Tables S1 and S2). The formation of non-native disulfide bonds by large scale *S*-thioallylation of protein thiols results in an impairment of protein homeostasis and a heat shock response, as shown also in *E. coli* [7]. Moreover, allicin caused the depletion GSH and protein thiols in *E. coli*, *S. aureus*, and eukaryotic cells [7,19,20,26].

Allicin and DAS4 are reactive sulfur species (RSS), and their degradation leads to formation of other RSS, such as allyl thiols, allyl persulfides, and H_2_S [3,5,62]. Thus, allicin and DAS4 might also cause induction of RSS-specific regulons, such as sulfur transferases. Interestingly, we noted the very strongly induced *yrkEFHIJ* operon, exhibiting log2-fold changes of 6.5–11 under allicin and DAS4 (Figure 2A,B, Tables S1 and S2). The *yrkE* gene encodes a putative sulfur transferase, the *yrkFJ* genes share homology to genes encoding sulfur carrier protein subunits of the TusA family, and *yrkJ* could encode a sulfonate uptake permease. Thus, this *yrkEFHIJ* operon could function as novel sulfur-specific uptake and degradation operon. This sulfur-specific operon is connected to the YrkP regulon consisting of the *yrkON,* *yrkPQR*, and *ykcBC* operons, which are also weakly up-regulated by garlic sulfur compounds (log2 fold change of 1–2). 

Apart from these thiol-stress responses, allicin and DAS4 caused the induction of the SOS response LexA regulon, revealing a DNA damage response in *B. subtilis*. Increased transcription of the SigD and SigM regulons in response to allicin and DAS4 stress was further noted. The SigD regulon controls motility and chemotaxis, and was previously induced under disulfide stress [27]. The SigM regulon was shown to respond to cell wall antibiotics, ethanol, heat, acid, and superoxide stress [63]. In addition, we noticed that the carbon catabolite control CcpA regulon responds moderately to allicin and more strongly to DAS4 stress, which could point to the utilization of allicin or its degradation products allyl thiols as alternative carbon sources (Figure 2A,B, Tables S1 and S2). Of note was particularly the *rbsRKDACB* operon, that encodes the ribose uptake ABC transporter with log2 fold-changes of >6 for *rbsA, rbsB*, and *rbsC*. In conclusion, allicin and DAS4 lead to a very similar thiol-specific oxidative, disulfide and sulfur stress response and protein damage in *B. subtilis*, which is comparable to the disulfide stress responses caused by other thiol-reactive compounds, such as diamide and HOCl. These transcriptomics signatures for allicin and DAS4 are in agreement with their main mode of actions to impair the thiol-redox homeostasis by *S*-thioallylation of LMW and protein thiols [7,9,19].

### 3.3. Allicin and DAS4 Lead to Widespread S-Thioallylation of Total 108 Proteins in B. subtilis 

**Figure 3:**
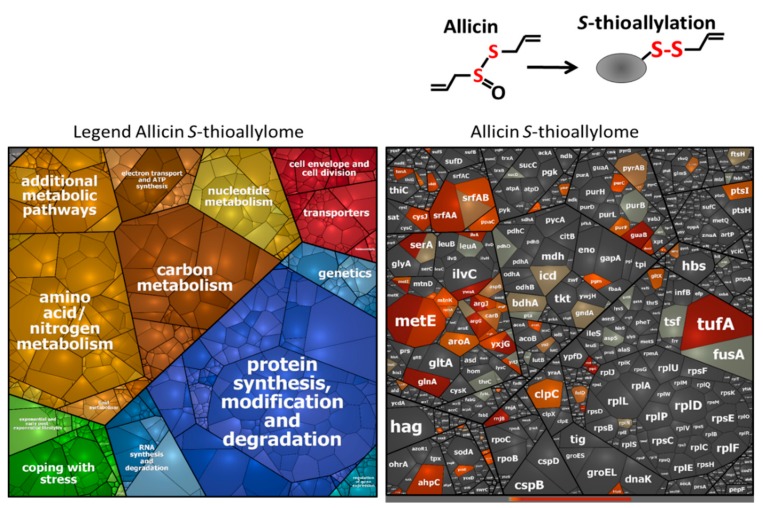
Allicin leads to *S*-thioallylation of 79 proteins in the *B. subtilis* proteome. Cytoplasmic protein extracts from allicin-treated *B. subtilis* cells were subjected to shotgun LC-MS/MS analysis for identification of 79 *S*-thioallylated proteins, which showed a mass increase of 72 Da at Cys residues. The treemap is based on protein abundances of all proteins identified and quantified in the proteome by spectral counting using the Scaffold proteome software. The cell size indicates the protein abundance. Proteins were allocated into functional categories based on TIGRfam annotation. *S*-thioallylated proteins are labelled using a red–grey color gradient based on the spectral counts of their *S*-thioallylated Cys peptides and are listed in Tables S3–S6. Among the most abundant *S*-thioallylated proteins by allicin are translation elongation factors (Tuf, Tsf, FusA), surfactin synthetase (SrfAA, SrfAB), and biosynthetic enzymes for amino acids (MetE, YxjG, MetI, AroA, ArgJ, CarB, GlnA) and nucleotides (GuaB, PpaC, PurB, PyrAB).

**Figure 4:**
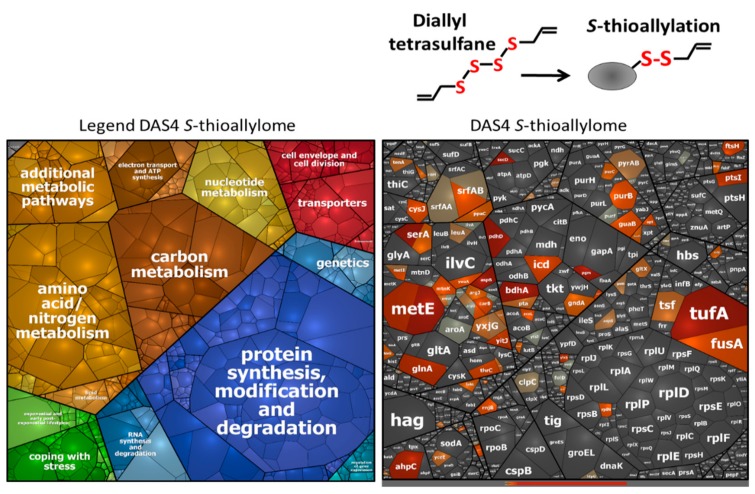
DAS4 causes *S*-thioallylation of 66 proteins in the *B. subtilis* proteome. Cytoplasmic protein extracts from DAS4-treated *B. subtilis* cells were subjected to shotgun LC-MS/MS analysis for identification of 66 *S*-thioallylated proteins. The treemap is based on protein abundances indicated by the cell sizes. Proteins were classified by TIGRfam annotations. *S*-thioallylated proteins are labelled using a red–grey color gradient based on the spectral counts of their *S*-thioallylated Cys peptides and are listed in Tables S3–S6. The most abundant *S*-thioallylated proteins by DAS4 include also translation elongation factors (Tuf, Tsf, FusA), surfactin synthetase (SrfAA, SrfAB), and biosynthetic enzymes for amino acids (MetE, YxjG, MetI, AroA, ArgJ, CarB, GlnA) and nucleotides (GuaB, PpaC, PurB, PyrAB).

Allicin has been previously shown to modify numerous cytoplasmic proteins by *S*-thioallylations in *E. coli*, *S. aureus* and human Jurkat cells [7,19,26]. Here, we were interested to identify the targets of *S*-thioallylation in the proteome of *B. subtilis*. We used Orbitrap LC-MS/MS analysis to investigate the *S*-thioallylome in *B. subtilis* after exposure to 90 µM allicin and 92 µM DAS4 stress. *S*-thioallylated proteins were identified by a mass increase of 72 Da at Cys peptides (Tables S3–S6). Both allicin and DAS4 resulted in a large extent of 108 *S*-thiolated proteins in the proteome. While 89 Cys residues in 79 proteins were *S*-thioallylated by allicin, DAS4 treatment resulted in *S*-thioallylation of 76 Cys residues in 66 proteins. The majority of 53 Cys residues in 44 proteins were modified by both allicin and DAS4. The *S*-thioallylated proteins were allocated to functional categories based on TIGRfam annotation in *B. subtilis*, such as information processing (e.g., transcription, protein synthesis), biosynthesis of amino acids, cofactors and nucleotides, energy metabolism, and adaptation to environmental changes (Table S4). The targets for *S*-thioallylations by allicin or DAS4 were color-coded in the Voronoi proteome treemap based on the abundance of detected *S*-thioallylated peptides using spectral counts (Figure 3 and Figure 4). 

In total, we could quantify about 1137 proteins using spectral counts with the Proteome software Scaffold in the proteome of allicin and DAS4-treated cells of *B. subtilis* (Table S5). The most abundantly *S*-thioallylated proteins under allicin and DAS4 stress were protein translation factors (TufA, FusA, Tsf), the surfactin synthetase subunits (SurfAA, SurfAB) and enzymes for amino acid biosynthesis, such as methionine (MetE, YxjG, YitJ, MetI, MtnA, MtnK), arginine (AspB, ArgG, ArgJ, ArgF, CarB), glutamine (GlnA), serine (SerA), and aromatic amino acids (AroA) (Tables S3–S6). Among these, TufA, FusA, and MetE were modified at their conserved Cys residues, e.g., Cys83 of TufA, Cys239 of FusA, and Cys719/Cys730 of MetE. This indicates that reactive garlic compounds target most strongly abundant thiol-containing proteins with conserved Cys-residues. Interestingly, TufA, FusA, and GlnA were also *S*-thioallylated at the same conserved Cys residues in the allicin proteome of *S. aureus* [19]. In addition, proteins involved in nucleotide biosynthesis, such as purine and pyrimidine biosynthetic enzymes (PurB, PurC, PurF, PyrAB), the IMP dehydrogenase GuaB, and the manganese-dependent inorganic pyrophosphatase PpaC were *S*-thioallylated by allicin and DAS4 in *B. subtilis*. GuaB and PpaC were previously identified as allicin targets in the proteome of *S. aureus* [19]. In *B. subtilis,* GuaB and PpaC were *S*-thioallylated at their conserved redox-sensing active sites Cys308 and Cys158, respectively. 

Overall, TufA, MetE, YxjG, GuaB, SerA, and PpaC were modified at their redox-sensitive Cys residues by allicin and DAS4, which were previously identified as targets for *S*-bacillithiolation under HOCl stress in *B. subtilis* (Tables S3–S6) [27,30,31]. The allicin and DAS4 targets included the peroxiredoxin AhpC and the methionine sulfoxide reductase MsrB, which were *S*-thioallylated at conserved Cys residues. In conclusion, the reactive sulfur compounds of garlic allicin and polysulfides modify largely conserved redox-sensitive active site Cys residues in the proteomes of *S. aureus* and *B. subtilis*. 

However, we did not find *S*-thioallylated redox-sensitive regulators in our proteome dataset using the shotgun proteomics approach, which might be related to their low abundance in the proteome. Based on our transcriptome results, allicin and DAS4 resulted in strong induction of the OhrR, HypR, YodB, and CatR regulons in *B. subtilis*, which are controlled by redox-sensing MarR/OhrR-type and MarR/DUF24 family regulators. Thus, we were interested whether these MarR-type repressors sense garlic compounds by *S*-thioallylation. We used immunoprecipitation to pull-down OhrR-FLAG protein with anti-FLAG agarose from cell extracts of allicin-treated *B. subtilis* strain HB9121. Allicin-treated cell extracts of *B. subtilis* 168 were used to pull-down HypR, YodB, and CatR proteins with polyclonal antibodies. 

Orbitrap mass spectrometry of the IP samples enabled the identification of conserved redox-sensing Cys residues as modified by *S*-thioallylation for OhrR (Cys15), HypR (Cys14), and CatR (Cys7) (Tables S3–S6). For the YodB-repressor, only Cys101 was *S*-thioallylated, but the *N*-terminal redox-sensitive Cys6 peptide could not be identified. Thus, our results confirm that the redox-sensitive MarR-type repressors OhrR, CatR, and HypR sense allicin by *S*-thioallylation, resulting in repressor inactivation and induction of the peroxidiredoxins (OhrA) and flavin disulfide reductases (HypO) that might function in allicin detoxification in *B. subtilis*. Similarly, the MarR/SarA family regulators MgrA, SarA, and SarS were previously shown to be *S*-thioallylated by allicin and MgrA was *S*-sulfhydrated at the redox-sensing Cys12 in response to H_2_S in the proteome of *S. aureus* [19,64]. Overall, we have identified 108 *S*-thioallylated proteins in the proteome of *B. subtilis* in response to allicin and DAS4 stress. These contain 42 Cys peptides with conserved Cys residues as revealed by the conserved domain database (Table S4). Thus, the main targets for *S*-thioallylations in *B. subtilis* are translation elongation factors (TufA, Tsf, FusA), redox-sensing MarR-type regulators (OhrR, HypR, CatR), many biosynthetic enzymes (GuaB, MetE, YxjG, SerA, PpaC), and antioxidant enzymes (AhpC, MsrB) that harbor conserved active site Cys residues and overlap strongly with the allicin targets in the proteome of *S. aureus* [19]. 

### 3.4. The LMW Thiol Bacillithiol and the Redox-Sensitive Regulators OhrR, HypR, and Spx Functions in the Defense of B. subtilis Against Allicin Stress

**Figure 5:**
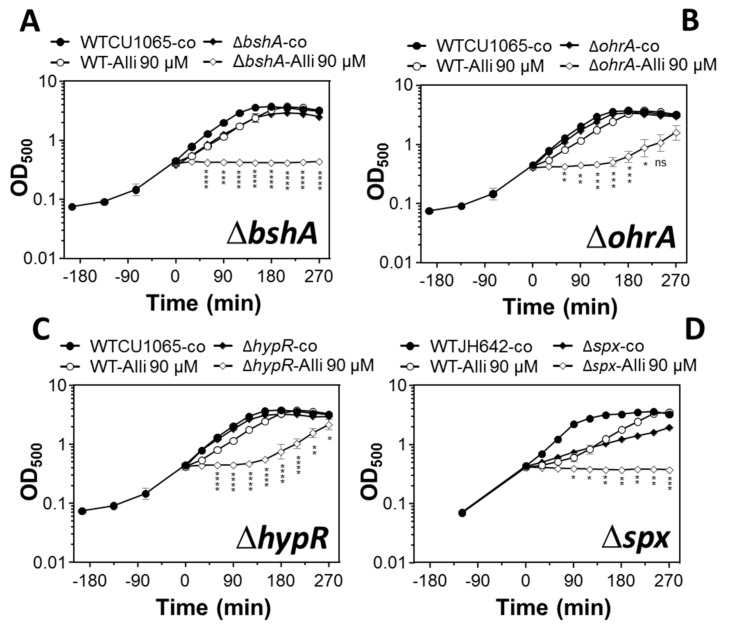
The *bshA*, *ohrA*, *hypR*, and *spx* mutants show growth defects under allicin stress in *B. subtilis*. Growth curves were monitored for the *B. subtilis* wild type (WT), the *bshA* (**A**), *ohrA* (**B**), *hypR* (**C**), and *spx* mutants (**D**) before and after treatment with 90 µM allicin stress. The strains were cultivated in BMM and exposed to allicin at an OD_500_ of 0.4 All mutants were impaired in growth under allicin stress. The results are from 3 biological replicates. Error bars represent the SD. The statistics was calculated using a Student’s unpaired two-tailed t-test by the GraphPad Prism software. The *p*-values are *p* < 0.0001 at 120–270 min in (A); *p* < 0.001 at 120–180 min in (B), *p* < 0.0001 at 60–210 min in (C), and *p* < 0.01 at 150–240 min in (D). Symbols are ^ns^ *p* > 0.05, * *p* ≤ 0.05, ** *p* ≤ 0.01, *** *p* ≤ 0.001, and **** *p* ≤ 0.0001.

Next, we were interested whether the LMW thiol BSH and the antioxidant enzymes controlled by redox-sensitive regulators OhrR, HypR, and Spx provide protection against allicin stress in *B. subtilis*. Thus, growth curves of the *bshA*, *ohrA*, *hypR*, and *spx* mutants were monitored compared to the wild type after exposure to sub-lethal 90 µM allicin stress. Allicin resulted in a short lag of growth for 60 min in the wild type, followed by fast resumption of growth with the same growth rate as untreated control cells. In contrast, the *bshA* mutant was impaired in growth after exposure to 90 µM allicin (Figure 5A). Treatment of the *ohrA* and *hypR* mutants with 90 µM allicin also led to a reduced growth rate and delayed resumption of growth after 180 min (Figure 5B,C). Thus, the LMW thiol BSH, the OhrR and HypR regulons provide protection against allicin toxicity in *B. subtilis*, possibly by detoxification of allicin. The *spx* mutant displayed a reduced growth rate even under non-stress conditions and was unable to grow with 90 µM allicin compared to the wild type (Figure 5D). These results indicate a role of the Spx regulon to combat thiol-stress provoked by allicin in *B. subtilis*.

## 4. Discussion

The antimicrobial and toxic effects of garlic can be attributed to the thiosulfinate allicin and diallyl polysulfanes of different sulfur chain length, which are generated during damage, heating, or aging of garlic tissues [5]. The antimicrobial mode of action of garlic-derived reactive sulfur compounds is mainly caused by its thiol-reactivity and depletion of the reduced thiol pool, including LMW thiols (GSH, BSH) and protein thiols. Allicin and diallyl polysulfanes react with LMW thiols via *S*-thioallylation, as demonstrated in the thiol-metabolomes of *E. coli*, *B. subtilis*, *S. aureus*, yeast, and human Jurkat cells [5,7,9,19,20,26]. However, the comparative mode of actions of allicin and polysulfanes as degradation products have not been studied in bacteria. It was further interesting to investigate whether allicin and polysulfanes modify similar protein targets by *S*-thioallylation. Thus, we used global transcriptomics and proteomics approaches to investigate in more detail the mode of action and targets for *S*-thioallylation in *B. subtilis* by both allicin and DAS4 in comparison. Using pulldown assays, we identified *S*-thioallylations at the redox-sensing Cys residues of the MarR-type repressors OhrR, HypR, CatR, and YodB as redox-sensing mechanisms under allicin stress leading to up-regulation of the corresponding redox regulons in the transcriptome. Phenotype analyses of mutants revealed potential roles of BSH, OhrA, HypR, and Spx in protection against allicin toxicity in *B. subtilis*. These results revealed new roles for the thiol-specific OhrA, HypR, and Spx regulons in protection under allicin stress.

Using microarrays, we could show that allicin and DAS4 cause a similar transcriptome signature, which is indicative of a strong oxidative, disulfide, and sulfur stress response in *B. subtilis*. The transcriptome comparison between the allicin and DAS4 responses in *B. subtilis* revealed similar high inductions of most thiol-stress response regulons, such as OhrR, PerR, HypR, Spx, CatR, YodB, and AdhR. In addition, allicin and DAS4 provoked a strong metal stress response and protein unfolding due to disulfide formation as shown by the induction of the ArsR, CzrA, CsoR, and CtsR regulons. Due to related transcriptome signatures, we can conclude that allicin and DAS4 show similar thiol-reactivities in *B. subtilis*. The allicin and DAS4 thiol-stress signatures overlap strongly with the transcriptome response of *B. subtilis* under HOCl stress, which causes *S*-bacillithiolation in the proteome [27,30,31]. In addition, allicin caused strong inductions of the related redox regulons controlled by HypR, PerR, QsrR, CsoR, and CstR in the RNAseq transcriptome of *S. aureus* [19], showing similar disulfide and metal stress responses as well as protein damage by allicin in both bacteria.

However, while the HypR-controlled flavin disulfide reductase *merA* gene was most strongly up-regulated under allicin and HOCl in *S. aureus* [19,58], the top scorer was the OhrR-regulated *ohrA* gene in *B. subtilis* under allicin, DAS4 and HOCl stress [27]. In *S. aureus*, we used NADPH coupled assays in vitro and phenotype analyses of the *merA* mutant in vivo to reveal an important function of MerA as allicin reductase as novel mechanism for allicin detoxification [19]. Since the *ohrA* mutant was impaired in growth under allicin stress, the peroxiredoxin OhrA could play a similar role in allicin reduction in *B. subtilis*. Moreover, the Spx and HypR regulators of *B. subtilis* control several putative thiol-disulfide oxidoreductases and flavoenzymes, which are highly up-regulated in the transcriptome (e.g., *nfrA*, *yugJ*, *ywcH*, *yjbH*, and *hypO*). In addition, *hypR* and *spx* mutants showed strong sensitivity towards allicin stress. These results suggest that HypR- and Spx-controlled oxidoreductases could also function in allicin detoxification, which remains to be investigated.

The decomposition of allicin and polysulfanes results in the formation of other RSS, including allyl thiols, allyl persulfides, and H_2_S [3,5,62]. Thus, the RSS-responsive CstR regulon was highly induced in the allicin transcriptome of *S. aureus* [19], including the *cstAB* operon that encodes for a thiosulfate sulfurtransferase and persulfide dioxygenase-sulfurtransferase [65,66,67,68,69]. In *B. subtilis*, no related RSS-specific detoxification mechanism is known. Interestingly, the *yrkEFHIJ* operon was highly induced by allicin and DAS4 in *B. subtilis* encoding for a sulfur transferase (*yrkE*) and TusA-like sulfur carrier proteins (*yrkFJ*) which could function in RSS detoxification. Overall, the transcriptome signatures of allicin and DAS4 indicate that both garlic compounds cause strong thiol-specific oxidative, metal, and sulfur stress responses, as well as protein damage in *B. subtilis* and *S. aureus* [19].

The protein damage response by allicin and DAS4 is caused by their *S*-thioallylation of protein thiols and LMW thiols, leading to depletion of the total cellular thiol pool. Widespread protein *S*-thioallylations have been mapped in previous studies and this study using shotgun proteomics in the *E. coli*, *S. aureus*, *B. subtilis*, and human Jurkat cell proteomes [7,19,26]. *S*-thioallylation of proteins leads to loss of protein functions as shown for selected targets, such as GapDH, cysteine protease papain, alcohol dehydrogenases, enolase, and isocitrate lyase in vitro [7,8,19,26].

In the *S. aureus* allicin proteome, 57 proteins were modified by *S*-thioallylations, which include mostly abundant proteins involved in protein translation (EF-Tu, EF-Ts, RpsB, RpmG2), biosynthetic enzymes for nucleotides, and amino acids (GlnA, AldA, GuaB) and antioxidant enzymes (KatA, Tpx, BrxA, MsrB) [19]. Among these, 37 proteins were modified at their redox-sensing active sites or conserved Cys residues indicating that garlic compounds target mainly redox-sensitive thiols [19]. In the *B. subtilis* allicin and DAS4 proteome, 108 *S*-thioallylated proteins were identified with similar functions in protein translation, the biosynthesis of nucleotides and amino acids, and detoxification of reactive oxygen species (ROS). These targets for *S*-thioallylations included 44 proteins that were modified at conserved Cys residues. Some redox-sensitive proteins are conserved targets for *S*-thioallylation by allicin in *B. subtilis* and *S. aureus*, such as Ef-Tu, EF-Ts, GlnA, GuaB, PpaC, MsrB, and GltX [19].

In addition, the targets for *S*-thioallylations under allicin stress overlap with those proteins *S*-bacillithiolated under HOCl stress in *B. subtilis* and *S. aureus*. These data support that garlic compounds target mostly redox-sensitive active sites [19,27,30,31,35,70,71]. The conserved *S*-thiolated proteins by HOCl and garlic disulfides are mainly metabolic enzymes, such as MetE, YxjG, GuaB, AldA, PpaC, and SerA, that are most likely redox-regulated and protected by *S*-thioallylation at their active site Cys residues [31]. In support of this, several redox-sensitive MarR-type regulators are among the main targets for *S*-thioallylation in both bacteria, such as SarA, MgrA, SarR, and SarH1 in *S. aureus* [19,72] and OhrR, HypR, CatR, and YodB in *B. subtilis*. Most of these MarR-type regulators were *S*-thioallylated at their redox-sensing Cys, leading to repressor inactivation (OhrR, CatR, YodB) or activation of HypR and up-regulation of the corresponding regulons. The redox-regulation of OhrR, HypR, CatR, and YodB due to *S*-thioallylation and induction of the corresponding regulons in the transcriptome was demonstrated in this study.

## 5. Conclusions

Altogether, our study revealed that allicin and DAS4 cause widespread *S*-thioallylation of many abundant proteins with conserved Cys residues, but also lower abundant redox-sensitive transcriptional regulators. Thus, *S*-thioallylation functions in thiol-protection and redox-regulation in bacteria to facilitate the recovery of growth and survival by induction of detoxification pathways. Our transcriptome and proteome studies further revealed that both allicin and DAS4 induce similar thiol-specific stress responses and hence exert similar thiol-reactivities on redox-sensitive Cys residues. Thus, allicin and polysulfanes can be applied as efficient thiol-reactive antimicrobials.

## Supplementary Materials

The following files are available online at https://www.mdpi.com/2076-3921/8/12/605/s1, Table S1: Transcriptome analysis of *B. subtilis* 168 wild type after Allicin and DAS4 treatment using microarrays, Table S2: Transcriptome analysis of *B. subtilis* 168 after exposure to Allicin and DAS4 stress and subsets of gene expression changes classified into regulons, Table S3: Identification of 108 *S*-thioallylated proteins in the *B. subtilis* 168 allicin and DAS4 proteome using shotgun LC-MS/MS analysis including their spectral counts, Sequest Xcorrs, deltaCn scores and mass deviations, Table S4: Functional classification based on TIGRfam of the 108 proteins with *S*-thioallylated Cys peptides identified in the *B. subtilis* proteome under allicin and DAS4 stress, Table S5: Total spectral counts of all identified proteins in the *B. subtilis* proteome under allicin an DAS4 stress, Table S6: Identification of 108 *S*-thioallylated proteins in the proteome of *B. subtilis* 168 under allicin and DAS4 stress using shotgun LC-MS/MS analysis and the spectral counts of identified *S*-thioallylated Cys peptides.

## Abbreviations

BSH	bacillithiol
Brx	bacilliredoxin
CHP	cumene hydroperoxide
GSH	glutathione
DAS4	diallyl tetrasulfane
H_2_O_2_	hydrogen peroxide
HOCl	hypochloric acid
LMW thiol	low molecular weight thiol
OD_500_	optical density at 500 nm
ROS	reactive oxygen species
RSS	reactive sulfur species
